# Two distinct and competitive pathways confer the cellcidal actions of artemisinins

**DOI:** 10.15698/mic2015.01.181

**Published:** 2015-01-02

**Authors:** Chen Sun, Jian Li, Yu Cao, Gongbo Long, Bing Zhou

**Affiliations:** 1 State Key Laboratory of Biomembrane and Membrane Biotechnology, School of Life Sciences, Tsinghua University, Beijing, China.

**Keywords:** heme, mitochondria, artemisinin, yeast, cancer

## Abstract

The biological actions of artemisinin (ART), an antimalarial drug derived from *Artemisia annua*, remain poorly understood and controversial. Besides potent antimalarial activity, some of artemisinin derivatives (together with artemisinin, hereafter referred to as ARTs), in particular dihydroartemisinin (DHA), are also associated with anticancer and other antiparasitic activities. In this study, we used baker’s yeast *Saccharomyces cerevisiae* as cellular and genetic model to investigate the molecular and cellular properties of ARTs. Two clearly separable pathways exist. While all ARTs exhibit potent anti-mitochondrial actions as shown before, DHA exerts an additional strong heme-dependent, likely mitochondria-independent inhibitory action. More importantly, heme antagonizes the mitochondria-dependent cellcidal action. Indeed, when heme synthesis was inhibited, the mitochondria-dependent cellcidal action of ARTs could be dramatically strengthened, and significant yeast growth inhibition at as low as 100 nM ART, an increase of about 25 folds in sensitivity, was observed. We conclude that ARTs are endowed with two major and distinct types of properties: a potent and specific mitochondria-dependent reaction and a more general and less specific heme-mediated reaction. The competitive nature of these two actions could be explained by their shared source of the consumable ARTs, so that inhibition of the heme-mediated degradation pathway would enable more ARTs to be available for the mitochondrial action. These properties of ARTs can be used to interpret the divergent antimalarial and anticancer actions of ARTs.

## INTRODUCTION

**Figure 1:**
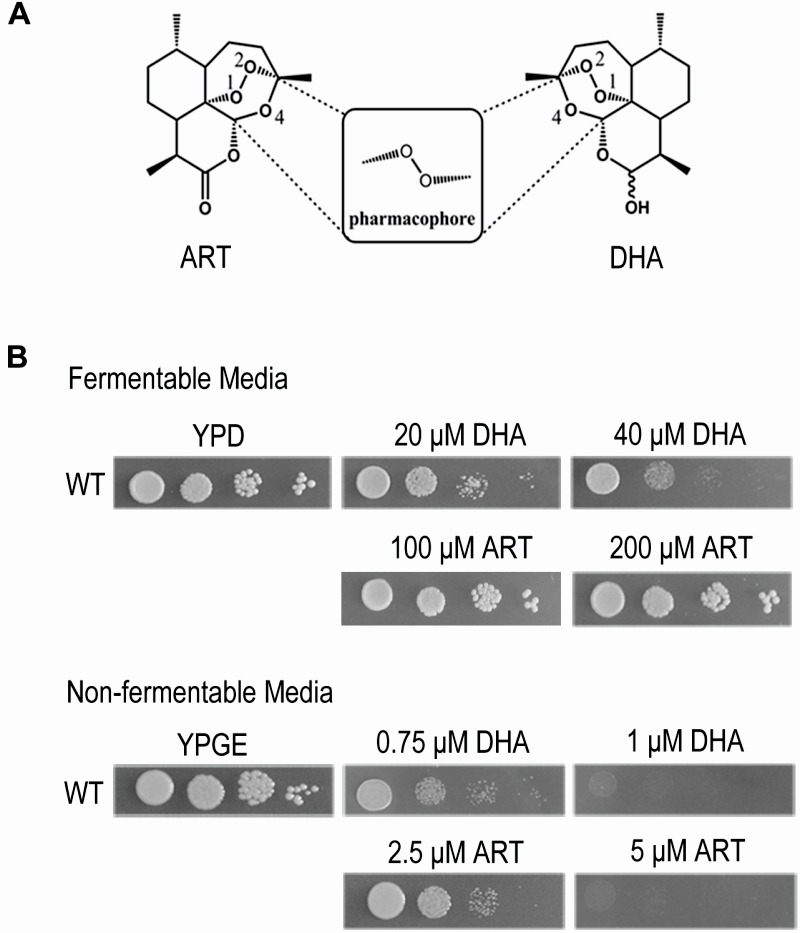
FIGURE 1: Dihydroartemisinin (DHA) inhibits yeast on both fermentable and nonfermentable media, albeit at much higher levels for the latter. Artemisinin (ART) has much weaker inhibition on the fermentable medium. **(A)** Molecular structures of ART and DHA. The endoperoxide bond in 1, 2, 4-trioxane is critical for the action of this class of drugs. **(B)** Inhibition of yeast growth on fermentable (YPD) and nonfermentable (YPGE) media with ART and DHA. DMSO was used when there was no drug added. ART: artemisinin; DHA: dihydroartemisinin.

Malaria is one of the most serious and prevalent infectious diseases worldwide. It remains a severe threat to human health; globally, an estimated 207 million cases of malaria and at least 627,000 deaths occurred in 2012 as reported by the World Health Organization (WHO) 1. In the early 1970’s, a large group of Chinese scientists, supported and organized by the Chinese government, extracted a highly effective and potent antimalarial drug, artemisinin (ART), from *Artemisia annua*, a traditional Chinese herb 2. The next 40 years witnessed the synthesis of a series of artemisinin derivatives (ARTs), including dihydroartemisinin (DHA, Fig. 1A), artesunate, artemether and so on 3,4,5,6. Due to the poor bioavailability of original ART, some artemisinin derivatives became more frequently used under clinical settings 7. Because of emerging drug-resistances against other antimalarial drugs such as quinine, chloroquine and atovaquone 8,9,10, ARTs are currently becoming our last defensive line against this devastating disease. For this reason, as well as the high recrudescence rate in ARTs-treated patients, WHO recommended ART-Combination Therapy (ACT).

Chemically, ART is a sesquiterpene lactone containing an endoperoxide bond, which is essential for its antimalarial action. Deoxyartemisinin, which contains the same backbone structure as ART but missing the peroxide bond, is about three orders of magnitude less effective than ART 11. Like H_2_O_2_, the peroxide bond in ART can be cleaved *in vitro* by ferrous iron, heme, or some other redox-active agents, similar to the Fenton reaction, to yield hydroxyl radicals. The majority of hypotheses concerning ART’s mechanism were proposed based on this reaction. Specifically, three different theories exist explaining the mode of action of ARTs 12: the protein target theory, heme theory and mitochondria theory. We previously established a yeast genetic model and found that ART interferes with mitochondrial function by depolarizing mitochondrial membrane potential 13. This action was later confirmed with purified mitochondria from yeast and malaria parasites 11. ARTs can quickly react with mitochondria of malaria parasites 11,14. In particular, ARTs directly induce ROS generation and membrane depolarization in purified yeast and malarial mitochondria, an effect that can be abrogated by ROS scavengers 11.

The specifics regarding anti-mitochondrial action of ARTs remain poorly understood. It was suggested that this anti-mitochondrial action may first involve ARTs activation by the electron transport chain (ETC), but direct evidence for this hypothesis is still lacking. We only know that ARTs’ action on mitochondria is not through a direct inhibition of ETC because electron transport/respiration is not repressed 11. Instead, the ETC may provide a reductive force for the activation of ARTs, which then interfere with mitochondrial functions 11,13. On the other hand, mammalian mitochondria do not activate ARTs, underlying the action specificity of these drugs. Therefore, although it is quite convincing that ARTs can be specifically activated by yeast and malarial but not mammalian mitochondria, leading to local damages and eventually cell death through resulting free radicals, the exact mechanism of mitochondrial activation of ARTs still remains uncertain and there is much that awaits to be explored.

In addition to the potent suppressing effect on malarial parasites, ARTs have also been reported to show inhibitory activities against other parasites 15,16, viruses 17 and also cancer cells 18,19. The potential usage of ARTs in cancer therapies has recently drawn much attention. ARTs, especially dihydroartemisinin (DHA) and artesunate, which acts through DHA, were shown to inhibit cancer cell growth at low μM levels *in vitro*; *in vivo* experiments, using mouse cancer models, also produced some promising results 20. Intensive efforts were devoted to exploring the mechanisms by which ARTs achieve the inhibitory effect against cancer cells. The cytotoxicity of ARTs against cancer cells was also demonstrated to be endoperoxide dependent 21. Moreover, modulating intracellular iron concentrations altered the effect of ARTs 22. The iron effect is consistent with the understanding that cancer cells contain more iron in order to maintain fast cell proliferation. Some recent studies further indicated that iron in the form of heme may be the mediator of the ARTs’ action 23,24. Regulating intracellular heme concentration of cancer cells altered the activity of ARTs correspondingly, while intracellular "free" iron did not directly participate in the action of ARTs but did increase heme synthesis 23.

Despite large efforts devoted to understanding how ARTs act biologically, it is still not known how exactly ARTs inhibit malarial parasites and cancer cells. In the past, medical chemistry studies helped reveal a large amount of *in vitro* properties of ARTs, but their biological properties in the context of a living cell were poorly understood. In order to decipher the biological actions of ARTs, we consider it essential to gain a comprehensive understanding of their molecular and cellular properties within a cell. In this report, we were able to reveal two modes of action of ARTs: the relatively nonspecific and general action as well as the mitochondrial action. We concluded that the heme-mediated nonspecific action and the specific mitochondrial pathway both exist *in vivo* and while both could be cell inhibitory, these two actions are also antagonistic likely due to their competition for the same source of the consumable ARTs.

## RESULTS

### High concentrations of ARTs, in particular DHA, can inhibit yeast growth even on fermentative media

**Figure 2:**
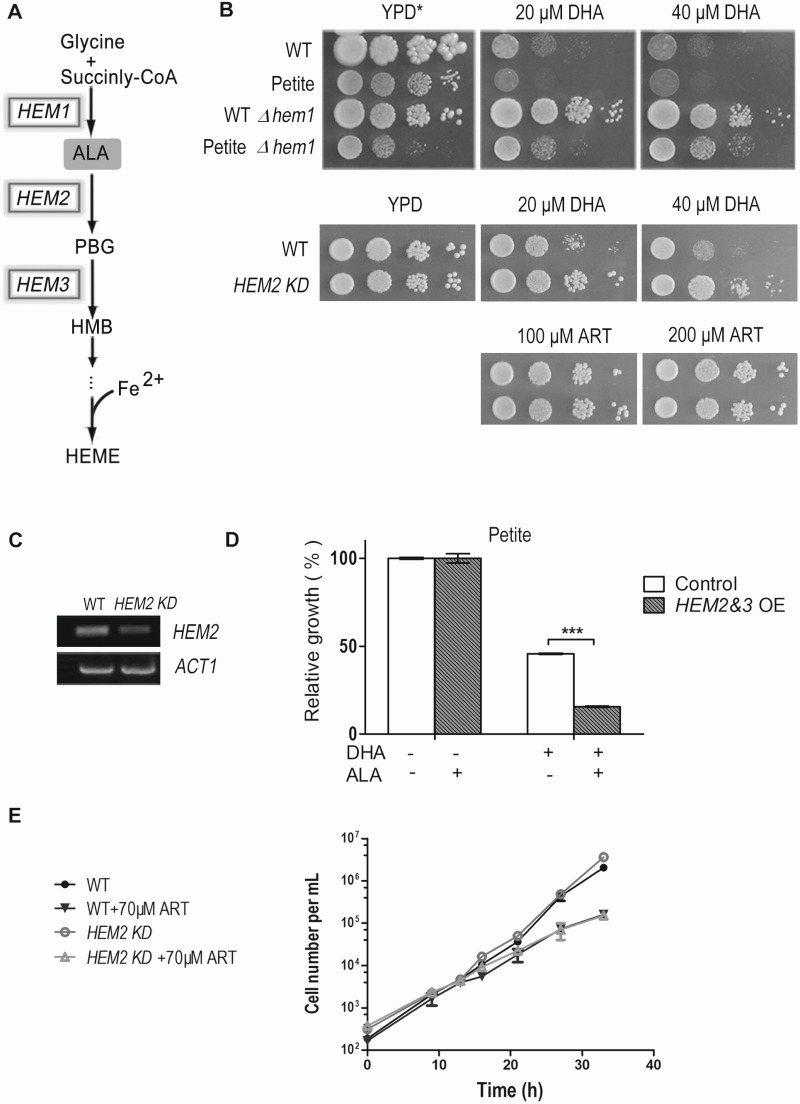
FIGURE 2: Heme mediates the inhibitory effect of DHA on fermentable media. **(A)** The heme biosynthesis pathway in *Saccharomyces cerevisiae*. **(B)** Deletion of *HEM1 *essentially eradicates DHA inhibition of yeast on fermentable media. YPD medium supplemented with Tween-80 and cholesterol (YPD*) was used. *HEM2*-knockdown (*HEM2-KD*) strain is also resistant to DHA on fermentable media. **(C)** RT-PCR analysis of RNA extracted from wild type and *HEM2-KD* strain**. **Semi-quantitative RT-PCR reveals a down-regulation at the mRNA level of *HEM2* in the *HEM2-KD* strain. *ACT1* is the control. **(D)** Overexpression of *HEM2&3*(*HEM2&3* OE) sensitizes yeast to DHA in the presence of δ-aminolevulinic acid (ALA). The effect was not as dramatic and could not be detected with the plate assay and thus a liquid growth assay was adopted. A petite strain was used. The grey bar represents *HEM2&3 *OE strain and white bar is the control strain with an empty vector. ***, P < 0.001. **(E) **The growth of the WT petite and *HEM2-KD* petite yeast in the presence and absence of ART in YPD liquid medium.* HEM2-KD* petite yeast is not more resistant than normal petite yeast to ART in fermentable media. Similar results were observed in 3 independent experiments.

Yeast is a facultative anaerobe and can grow on fermentable media (such as YPD medium wherein glucose is used as the carbon/energy source) and nonfermentable media (such as YPGE medium wherein ethanol and glycerol are used as the carbon/energy source) by either anaerobic or aerobic pathways. In the absence of mitochondrial respiration yeast cells adopt the anaerobic pathway and grow. This property makes it an ideal model organism to study the anti-mitochondrial properties of drugs. Our previous work demonstrated that yeast grown on nonfermentable media is a suitable model to study the mechanism of ARTs’ actions on mitochondria, and showed that ART efficiently inhibits yeast growth on nonfermentable media, but not on fermentable YPD medium even at a 10-fold increased concentration 13. At levels further up, inhibition by ARTs, in particular by DHA (Fig. 1A), on fermentable media becomes noticeable. As shown in Figure 1B, DHA can clearly inhibit yeast on YPD when about tenfold increase of drug concentration was applied. DHA is a more powerful antimalarial drug than ART, displaying about 5-fold higher antimalarial potency 11. Consistently, it is also more powerful in inhibiting yeast growth on nonfermentable YPGE medium. In comparison to the strong suppression of 5 μM ART on normal yeast growth on YPGE plates, 1 μM DHA exhibited a similar level of inhibition. On fermentable YPD medium, growth inhibition was obviously observed when 20 μM DHA was used, but little growth inhibition was seen even when 200 μM of ART (more than ten times as used on nonfermentable media such as YPGE) was added to YPD plates (Fig. 1B). The much less potent and arguably insignificant inhibition by ART in fermentable media could be observed when growth in liquid media was monitored 13 (and also see below, Fig 2E). One thing worthy of mentioning is that although liquid inhibition is theoretically more quantitative, in reality we found using plate assays to examine the inhibition of ARTs on yeast growth not only more convenient, but also more reproducible.

**Table 1:** Heme content of yeast strains grown on different media. *ng/mg protein Heme content was measured by a microplate reader (Fluoroskan Ascent) with excitation at 400 nm and emission at 660 nm. All of the results are expressed as the means ± S.D. calculated from four independent experiments.

	**YPD**					**YPGE**			**SD-Ura-Trp**			**SGE-Ura-Trp**	
**Strains**	**WT**	**Petite**	**WT Δ*hem1***	**Petite Δ*hem1***		**WT**	***HEM2*-KD**		**Petite**	**Petite*HEM2*&*3 *OE**		**WT**	**WT*HEM2*&*3 *OE**
Heme content*	7,2 ± 1,19	8,95 ± 1,11	1,06 ±0,73	1,05 ±0,46		23,03 ± 1,86	9,83 ± 0,49		11,03 ± 0,74	44,56 ± 4,26		22,88 ±8,10	104,42 ±26,39

In the yeast liquid growth assay for ARTs, the amount of cells inoculated, incubation duration and likely other factors dramatically affect the final result, possibly because ARTs are highly degradable, thus decreasing the actual drug concentration and significantly deviating it from the initial or designed one. When performed side by side and with well-controlled cell numbers and time, experimental errors are within a limited range. However, experiments performed at different times and under different conditions, can produce dramatically different absolute numbers (such as IC50), although with similar trends. In the plate assays, cell numbers inoculated are usually small and other likely contributing factors such as aeration might be less variable, making the assay more stable. We thus routinely used plate assays and resorted to well-controlled liquid assays, which are more quantitative but as explained above deliver relative and not absolute results, only when little difference could be noticed on the plate assays.

While DHA is somewhat more active than ART on nonfermentable media, it is much more effective than ART on fermentable media. This divergence of correlated activities for ART and DHA was reminiscent of findings found in cancer cell inhibition: while DHA can inhibit some cancer cells (IC50 normally in the range of a few to several dozens of μMs), ART is much less effective 25.

These results, in particular the inhibitory action of ARTs on yeast utilizing fermentable carbon sources, suggest to us the existence of a different and less potent type of inhibition besides the anti-mitochondrial action.

### Heme mediates DHA’s inhibitory action on fermentable media 

Previous studies have shown that intracellular heme physiologically mediates the cytotoxicity of some ARTs in tumor cells 23,24. Direct interaction between heme and ARTs, as reported in numerous *in vitro* studies 26,27, is well accepted in the medical chemistry field. To investigate the underlying mode of DHA’s action on fermentable media, we tested whether DHA’s inhibition on YPD medium can be affected by modulation of heme levels or not. Heme is indispensable for yeast growth on both fermentable and nonfermentable media. However, on fermentable media, heme requirement can be bypassed by the supplementation of Tween-80 and cholesterol to the medium, whose cellular synthesis presumably necessitates heme’s presence 28. Loss of function of *HEM1,* which catalyzes the first step of the heme synthesis pathway, drastically reduced heme synthesis (Fig. 2A, Table 1). In the conditional medium, which enables the growth of Δ*hem1,* the DHA inhibition on yeast was essentially completely eradicated (Fig. 2B). To be certain this was free of anti-mitochondrial action, we also examined whether petite strains, which lack respiration-functional mitochondria, behave similarly.

DHA can similarly inhibit petite yeast in a heme-dependent manner (Fig. 2B). We then wondered whether up-regulating heme synthesis would show the opposite effect. With this consideration, we overexpressed the rate-limiting enzymes of heme synthesis 29 and supplemented this with the precursor/substrate δ-aminolevulinic acid (ALA). The effect due to heme increase was not as obvious as in the case of heme knockdown and was not noticeable on plate assays, so we had to use liquid growth to monitor the inhibition difference. In the liquid assay, overexpression of *HEM2&3* or supplementation of ALA into the medium alone did not obviously alter DHA sensitivity. However, when combined together, an apparent increase of DHA sensitivity was observed (Fig. 2D). Consistent with the improved inhibition efficacy, heme content was dramatically upregulated after the combined treatment (Table 1).

A *HEM2*-knockdown strain (*HEM2-KD*) was also utilized, wherein a KanMX4 coding sequence was inserted to the 3’ end of *HEM2* gene 30. This insertion leads to instability and degradation of *HEM2* mRNA, resulting in decreased heme synthesis (Table 1). RT-PCR indicated that the *HEM2* mRNA level was indeed down-regulated (Fig. 2C). Results showed that *HEM2-KD *yeast was significantly more resistant to DHA on fermentable media (Fig. 2B). We also tested whether heme is involved in ART’s inhibition in fermentable media. As shown previously, ART has only weak inhibition of yeast in fermentable media. While DHA’s non-mitochondrial effect is much affected by heme levels (Fig. 2B), *HEM2-KD* petite yeast is not more resistant than normal petite yeast to ART in fermentable media (Fig. 2E). This indicates that the much milder non-mitochondrial inhibition by ART, as observed in fermentable media, is not mediated by heme. The underlying mechanism for the difference of inhibitory properties between DHA and ART remains to be explored, nevertheless it is beyond the scope of this investigation.

### Heme antagonizes ARTs’ action on nonfermentable media

**Figure 3:**
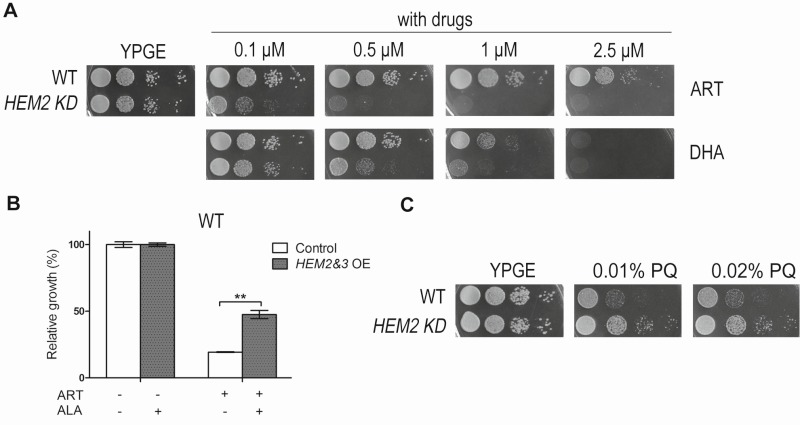
FIGURE 3: Modulation of heme synthesis dramatically alters the ART potency on nonfermentable media. **(A)** *HEM2-KD* strain is much more sensitive to both ART and DHA on YPGE medium. **(B)** ALA increases the resistance of *HEM2&3* overexpression (*HEM2&3* OE) strain to ART in nonfermentable media. Dropout medium SGE-Ura-Trp was used here. The grey bar represents *HEM2&3 *OE strain and white bar is the control strain with an empty vector (**, P < 0.01). **(C)** While more sensitive to ARTs, *HEM2-KD *yeast is slightly more resistant to paraquat (PQ), a mitochondria ROS generator.

Having shown that heme underlies DHA’s action on fermentable medium, we next asked whether heme is in any way related to the mitochondrial mode of action of ARTs. ARTs are all potently cell-inhibitory in nonfermentable media 11 (Fig. 3A). Because heme is a component of ETC, complete removal of heme would cause respiration-defective phenotype. In fact, higher heme content was detected when yeast was grown on nonfermentable medium YPGE as compared with that on fermentable medium YPD (Table 1). To investigate how heme is related to the anti-mitochondrial action of ARTs, a *HEM2*-*KD* strain was utilized. Results of spotting assays are shown in Fig 3A. To our surprise, heme strongly antagonizes the mitochondrial mode of action. *HEM2-KD* yeast is much more sensitive to ARTs: whereas normal yeast is inhibited by 1 μM DHA on nonfermentable medium (YPGE), the growth of this heme synthesis reduction strain was greatly inhibited on YPGE with much lower DHA concentrations. Worth noting is that this potentiating effect is even more apparent for ART: on nonfermentable media (YPGE), the growth of this *HEM2-KD* strain was greatly inhibited with 0.1 μM ART as compared to about 5 μM ART for the normal isogenic control strain (Fig. 3A). In contrast, when heme levels were upregulated by the overexpression of *HEM2&3* in addition to ALA, cell resistance to ART was increased on nonfermentable media (Fig. 3B). Again, this difference was less robust and we had to resort to liquid growth assays to detect the increase of drug resistance. Nevertheless, these results enabled us to conclude that heme plays a negative role in the mode of action of ARTs on nonfermentable media.

### Heme reduction strengthens ARTs’ anti-mitochondrial effects

**Figure 4:**
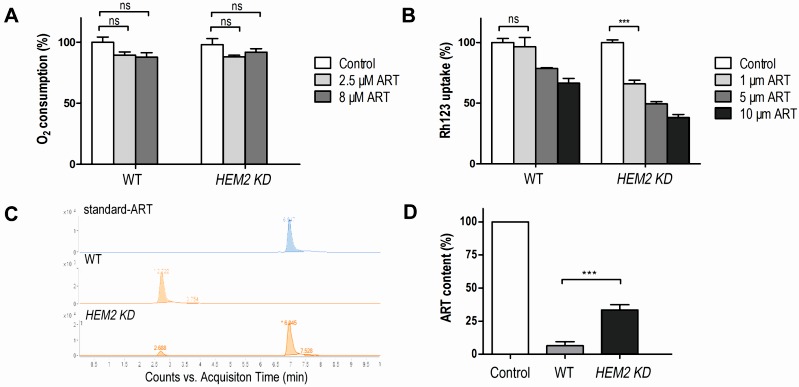
FIGURE 4: Heme reduction strengthens ARTs’ anti-mitochondrial effect. **(A)** ART had little negative effect on O_2 _consumption in both the WT and *HEM2-KD* strains (ns, no significance). **(B) **Similar levels of mitochondrial depolarization at much lower ART concentrations could be observed when heme is down-regulated. The membrane potential ( ΔΨm ) was assessed by measuring the ΔΨm-dependent uptake of Rh123. Lower uptake of the probe indicates lower ΔΨm. **(C, D)** *HEM2-KD* yeast has reduced ART’s metabolism rate as compared to WT. **(C) **is the chromatogram of ART in LC-MS analysis. The peak of ART is at 6.94 min. **(D) **is quantitative measurement of results from three parallel experiments. ***, p<0.001

The potentiating effect of heme down-regulation on ARTs’ inhibitory activity in nonfermentable but not in fermentable media suggests that heme antagonizes ARTs’ anti-mitochondrial action. However, because heme is also central to ETC activity in respiration, the increased sensitivity of *HEM2-KD* yeast to ARTs could alternatively be explained by the depleting effect of ARTs on heme in the ETC. In other words, it could be argued that when heme is down-regulated, heme in the ETC may be more vulnerable to the depleting effect of ARTs and therefore confers the increased sensitivity. If this holds true, we would expect that an addition of ARTs would reduce the ETC/respiration activity of yeast cells. We therefore tested the respiration ability of WT and *HEM2-KD* strains in nonfermentable medium YPGE with or without ART incubation. ART had little negative effect on O_2_ consumption in both the WT and the heme-limited strain, indicating that the heme participating in respiration was not affected by ART (Fig 4A). Meanwhile, the mitochondrial membrane potential (monitored by rhodamine 123 uptake) of WT and *HEM2-KD* yeast strains were both greatly reduced upon ART treatment (Fig 4B). These results indicate that the potentiating effect of heme limitation on ARTs is not through affecting the heme in the ETC.

We observed similar levels of mitochondrial depolarization at much lower ART concentrations when heme was down-regulated (Fig. 4B). Therefore, heme limitation greatly strengthens ARTs’ anti-mitochondrial effects.

ARTs’ action is related to ROS generation. Is it possible that heme limitation increases ARTs’ anti-mitochondrial potency because of a likely ROS scavenging activity of heme? To test whether heme could be a significant mitochondrial ROS scavenger *in vivo*, we tested whether heme down-regulation could increase the sensitivity to paraquat, a ROS generator in mitochondria 31. *HEM2-KD *strain was found not more sensitive to paraquat, if anything, slightly more resistant to this agent (Fig. 3C) on nonfermentable medium YPGE, indicating that heme reduction possibly does not result in a significant loss of ROS scavenging activity.

### Heme limitation reduces ARTs’ intracellular consumption/metabolism rate 

*In vitro* reactions of ARTs with non-heme or heme iron have been reported. For example, by using high-performance liquid chromatography (HPLC), heme-artemisinin adducts were isolated in a test tube reaction 32, and the physiological relevance of the interaction between heme and ART was further implied 33. The capability of ART to inhibit the proteolytic activity of digestive vacuoles was observed in both *ex vivo* and *in vitro* experiments in which ART could potently inhibit heme polymerization 34; ART was also demonstrated to be able to alkylate heme in infected mice 35. We figured that the potentiating effect of heme limitation on ARTs’ anti-mitochondrial action might be through an increase in the drugs’ availability as a result of reduced consumption of ARTs. Indeed, heme levels play an important role in the metabolism of ARTs. After incubation with 10 μM ART in nonfermentable YPGE medium for 20 min, LC-MS analysis indicated that the ART in the medium was virtually all metabolized during wild type yeast growth, suggesting a rapid consumption. On the contrary, the remaining ART level in *HEM2-KD* yeast medium was much higher and readily detectable (Fig. 4C and 4D). We explain this by the fact that ART can quickly transverse cell membranes so that rapid reduction of ART inside the cell can translate to rapid decrease of ART in the medium. *HEM2-KD* cells degrade ART more slowly so that depletion of ART in its growth medium takes longer. Indeed, it has been shown before that ART is enriched in most, if not all, cell membrane systems 36,37, and we and others have shown that ART addition to intact cells or mitochondria can result observable effects within minutes 11,14. These all support that artemisinin can fast transverse membranes. Our results therefore indicated that ARTs are depleted more slowly when the heme level is lowered, making heme a major player in the ARTs metabolism, i.e. the process of ART reduction and subsequent steps that are not yet well characterized.

### The heme-mediated inhibitory action of ARTs is not through interference of the iron-sulfur cluster biogenesis

**Figure 5:**
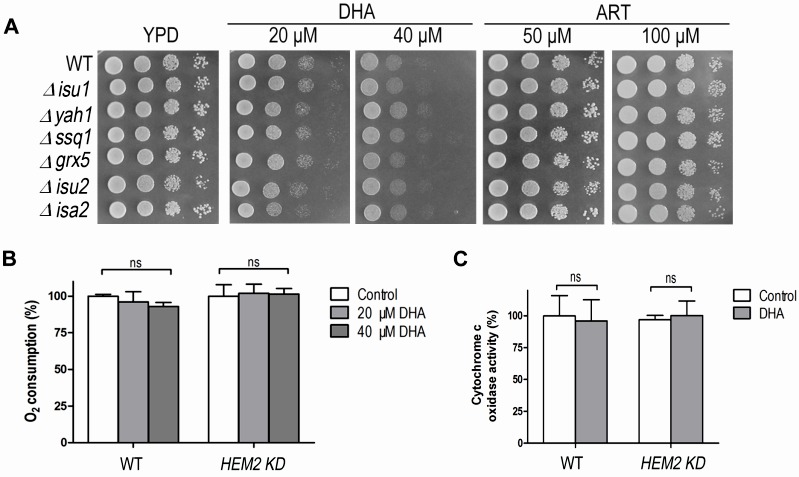
FIGURE 5: The heme-mediated inhibitory action of ARTs is not through interference of the iron-sulfur cluster biogenesis. **(A)** Compared with WT, the iron-sulfur cluster mutants do not exhibit extra sensitive phenotypes to both ART and DHA. All strains are in the BY4742 background. **(B)** DHA does not affect the respiration ability of either WT or *HEM2-KD *strains on fermentable media. **(C)** DHA does not affect the cytochrome *c* oxidase activity of either WT or *HEM2-KD* yeast strains. ns, no significance.

To investigate whether the heme-mediated suppression effect could be related to iron-sulfur cluster biogenesis, we tested several mutants with impaired iron-sulfur (Fe-S) protein biogenesis for their DHA sensitivity. Isu1p and Isu2p act as scaffold proteins for the Fe-S cluster assembly; Isa2p is involved in the [4Fe-4S] transfer to mitochondrial target proteins; mitochondrial chaperone protein Ssq1p as well as a small glutaredoxin-like protein Grx5p, participate in the transfer of the different types of Fe-S cluster from Isu1p/Isu2p scaffold to apoproteins; Yah1p is a [2Fe-2S] mitochondrial ferredoxin involved in heme A biosynthesis and in Fe-S cluster assembly 38,39. As shown in Fig. 5A, these mutants do not exhibit extra sensitive phenotypes to DHA on fermentable medium compared to WT. To further examine whether DHA affect iron-sulfur protein functions, respiration ability and cytochrome *c* oxidase activity were assayed. Cytochrome *c* oxidase is an iron-sulfur protein and it is reported that the cytochrome *c* oxidase activity decreases upon the depletion of components of the iron-sulfur cluster assembly machinery 38. However, no significant difference could be found in either the WT or heme knockdown strain with or without DHA incubation (Fig 5B, 5C), suggesting that the DHA toxicity in the fermentative growth condition is not due to the damage to iron-sulfur cluster biogenesis or inhibition of its functions.

## DISCUSSION

**Figure 6:**
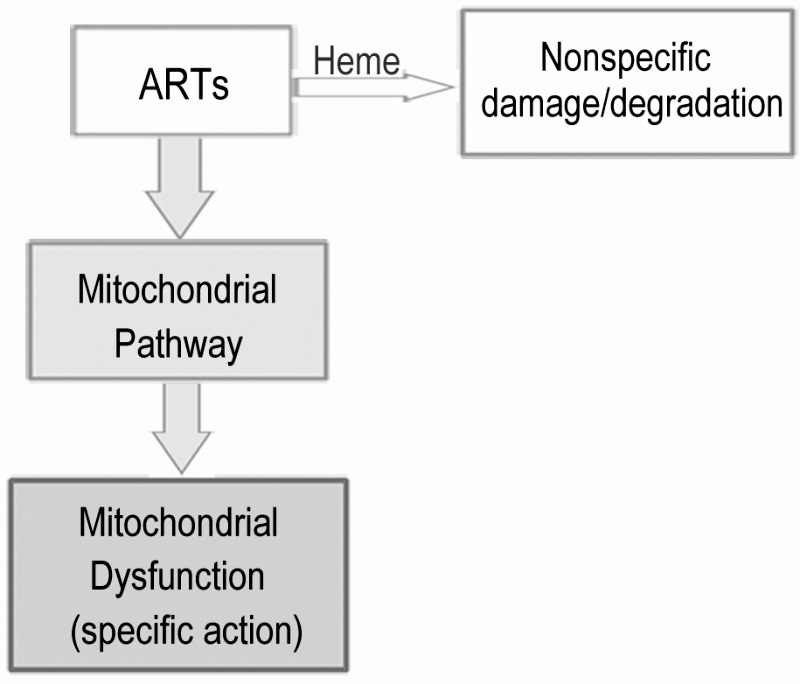
FIGURE 6: A model for the two distinct pathways of ARTs’ actions. ARTs act in two distinct ways: the heme pathway and the mitochondrial pathway. ARTs can be consumed by intracellular heme, inducing general damages (in the case of DHA) or tolerated/wasted (ART). ARTs can also be activated by the mitochondrial pathway, causing mitochondrial dysfunction. The heme pathway is a much less efficient pathway for killing and explains the action in some tumor cells and probably other general cell types. The much more potent mitochondrial pathway is shared by ARTs, and can explain the action of ARTs on malaria parasites and yeast grown on nonfermentable media.

The mode of action of ARTs has long been an intensely debated subject. In the present report, we used the yeast model to explore the biological properties of ARTs. We discovered the clear existence of two major types of pathways for ARTs’ actions: the heme-mediated and non-heme-mediated cell inhibitory actions. On fermentable media, upregulating and downregulating intracellular heme content could respectively increase and decrease sensitivity of yeast to DHA. On the contrary, when ARTs’ activity was assayed on nonfermentable media, which requires the mitochondrial respiratory function, modulating heme levels alters ART sensitivity in an opposite manner. Collectively, we propose a model of action for ARTs as illustrated in Fig 6. On one hand, ARTs can react with and be consumed by heme; on the other hand, ARTs can be activated by some unknown components (likely that of ETC) in mitochondria of certain organisms such as *S. cerevisiae* or malarial parasites. These pathways consume the same source of ARTs inside the cell. Between these two kinds of actions, the mitochondrial inhibition requires far less amounts of drugs and is greatly enhanced when the "wasting" pathway of heme is attenuated. Interaction of ARTs, in particular DHA, with heme likely generates nonspecifically ROS and some toxicity, underlying the mode of action of heme-dependent general cytotoxicity.

In our initial screen for ARTs that showed inhibitory activities against yeast cells on YPD medium, we tested several ARTs including ART, DHA, artelinic acid and artesunate, which are all potent antimalarial drugs. Except for DHA, which could suppress yeast growth at moderate concentrations, the other three drugs had significantly reduced effects. Interestingly, DHA also stands out among ARTs in inhibiting cancer cell growth 20,40. The divergence between antimalarial and anticancer potency and the correlated activity of ARTs on yeast in fermentable media and mammalian cell inhibition, from another perspective are consistent with the existence of two pathways of action for ARTs. Notably, yeast and cancer cells were reported to share many features, in particular, the "aerobic glycolysis", also known as the "Warburg effect". Likewise, comparative studies in previous and current works also found a high degree of correlation in cell inhibitory activities of these ARTs between yeast grown on nonfermentable media and malarial parasites 11.

However, while both ART and DHA interact with heme, we are not sure why ART is far less toxic on fermentable medium YPD. One possibility might be that the resulted ART products are less cytotoxic. Another possibility is that DHA and ART have different chemical properties in terms of water solubility, affecting their intracellular distributions. Alternatively, their reaction profiles with heme could be different. The subtlety of biochemical/biophysical differences underlying their differing biological properties is certainly an issue worthy of future investigation.

Previous reports suggested that mitochondria were involved in the action of ARTs against cancer cells via apoptosis in a mitochondria-dependent manner 41,42. Recently, a detailed study further pointed out that active mitochondria are responsible for ARTs-induced cytotoxicity since HeLa rho^0^ cells were more resistant to this class of drugs 24. However, this role of mitochondria was not due to their reductive activation of ARTs to carbon-centered radicals, and indeed the same extent of activation took place in both the wild type and rho^0^ cell lines. The mitochondrial involvement in cancer cell inhibition by ARTs is therefore likely an indirect one and secondary to its involvement in apoptosis induction.

The role of the hemoglobin degradation pathway for the action of ARTs in malarial parasites has been intensively studied 35,43,44. Heme generated during hemoglobin catabolism is trapped in the vacuoles and has been proposed to be either the target 45 or the activating agent 46. However, *Plasmodium falciparum* infecting alpha-thalassemic erythrocytes is resistant to ARTs *in vitro*, and heme and heme-containing proteins in erythrocyte are proposed to reduce ART’s effectiveness 47,48. Furthermore, when malarial parasites are cultured under CO, which can block the interaction of ARTs with heme, inhibition by ARTs is more effective than when malarial parasites are cultured in the presence of O_2_. Thus, the role of heme may be associated with degradation instead of activation of ARTs aiding in the killing of parasite 49. Our results, showing that reduced intracellular heme content on nonfermentable media potentiated the effect of ART while increased heme levels attenuated the activity, provide supporting evidences that if there is any involvement of the hemoglobin catabolism pathway in the action of ARTs in malarial parasites, it likely acts more as a competing pathway for the consumption of ARTs. This is consistent with the malarial studies concluding that heme plays a deactivating role 47,48.

Besides the heme molecules that are generated by the hemoglobin degradation and condensed/trapped in the vacuoles, malarial parasites also have a functional endogenous heme pathway, which might provide the synthesis of heme for the general need of the organisms. To date, the involvement of the endogenous heme pathway in the action of ARTs has not been clarified. In our preliminary experiments we found that neither inhibition of heme synthesis by succinylacetone, a *hem2* inhibitor, nor addition of ALA much altered the sensitivity of *Plasmodium falciparum* to ARTs, suggesting that the endogenous heme was not significantly associated with ARTs’ activities. We suspect the activity of the endogenous heme pathway is rather weak so that not much heme is *de novo* synthesized.

Taken together, the yeast cell studies reveal to us two distinct types of biological actions for ARTs: both the mitochondrial and heme-dependent nonspecific pathways can mediate ARTs’ damage. Emerging from our studies is this scenario: ARTs can react with a few components (largely heme or heme related) inside a cell, conferring a relatively general and nonspecific action in most, if not all, cells, and sometimes causing the general toxicity or side effects of the drugs; the mitochondrial interaction only occurs in those ARTs-sensitive cells. Our studies thus reconcile heme and mitochondria theories and further dissect their individual roles in the mechanism of action of ARTs. We suggest that the heme pathway could explain the antitumor properties of DHA, whereas the specific and potent mitochondrial pathway can explain the antimalarial action. The information provided by this study therefore not only helps us understand the biological properties of ARTs, but may also open a new avenue for anticancer ARTs designs. In addition, degradation by heme should also be a concern for new antimalarial endoperoxide drug development.

## MATERIALS AND METHODS

### Chemicals

ART and DHA were purchased from Chengdu Okay Medicine Co., Ltd (Chengdu, China). Hemin and ALA were purchased from Sigma. ARTs and hemin were prepared in DMSO, ALA was prepared in double distilled H_2_O (ddH_2_O). All the aliquots were used immediately or stored at -20°C for later use.

### Yeast strains and growth

Standard yeast media and growth conditions were used. *Saccharomyces cerevisiae* BY4742 was used as the wild type control (BY4742: *MATα his3*Δ*1 leu2*Δ*0 lys2*Δ*0 ura3*Δ*0*). Petite strains in BY4742 background were made by ethidium bromide treatment. Δ*hem1* and *HEM 2&3* overexpression strains for both wild type and petite strain, and the *HEM2*-*KD* strain were all kept in our lab and were based on the BY4742 background. Yeast was normally plated on YPD (2% glucose as carbon source) or YPGE (2% glycerol plus 2% ethanol as carbon source) agar plates supplemented with different concentrations of ART or DHA, and with 0.5% Tween-80 and 30 μg/ml cholesterol when necessary. For overexpression strains, uracil and tryptophan dropout synthetic medium was used.

### Drug sensitivity/resistance assay

For growth testing on agar plates, yeast previously grown on YPD plates was 10-fold serial diluted with sterile ddH_2_O, and then spotted on YPD or YPGE plates with or without drugs (Spotting assay). For tests in liquid media, yeast was cultured overnight in synthetic dropout media as follows: after being spun down and washed with ddH_2_O, cells were resuspended with either SD-Ura-Trp or SGE-Ura-Trp medium to an initial A_600_ of 0.01; cells were then cultured with or without ALA (final concentration 250 μg/mL), DHA (0.5 μM) and ART (5 μM). A_600_ was measured after 36 hours. Incubation was all performed at 30°C.

### Heme content determination

Heme content was measured as previously described 50. Briefly, 20 ml yeast cultures grown in different media (YPD, YPGE, SD-Ura-Trp or SGE-Ura-Trp) were harvested at the exponential phase, washed two times with ddH_2_O and resuspended in 150 μl extraction buffer (PBS plus protease inhibitor cocktail). After the addition of isometric glass beads, the mixtures were vortexed for 15 minutes and centrifuged at 15,000 g for 30 min at 4°C. The supernatant was transferred to a new microcentrifuge tube, and the yeast’s total protein concentration was measured by a BCA protein assay kit (Thermo Scientific). To prepare samples for a heme content assay, an aqueous oxalic acid solution (500 μl, 2 M) was added to each sample. The samples were mixed thoroughly before boiling for 30 min. Standard solutions of hemin (0.01 - 10 mM) were prepared in water/methanol (1:1, v/v) and boiled with oxalic acid as described above. These standard solutions and samples (200 μl) were then added into a 96-well plate and fluorescent intensities were measured by a microplate fluorometer (Fluoroskan Ascent) with the excitation wavelength setting at 400 nm and emission at 662 nm. Results were corrected by preparing samples without boiling to eliminate the influence of endogenous non-heme porphyrins.

### Yeast mitochondria isolation

Mitochondria were purified from yeast cells by Zymolyase digestion and differential centrifugation. The preparation was performed as previously described with little modification 51. Cells were collected and homogenized in a buffer containing 5.0 mM HEPES, 70 mM surcose, 0.22 M mannitol, proteinase inhibitor cocktail, 0.2% (wt/vol) BSA, pH 7.2 at 4°C. To remove nuclei and large membrane fragments, the homogenate was first centrifuged at 900 g for 10 min, and the resulting supernatant was subsequently centrifuged at 10000 g for 10 min to obtain crude mitochondrial fractions. The mitochondrial fractions were further purified by a sucrose gradient.

### Membrane potential determination 

The membrane potential of yeast cells was determined by measuring the fluorescence intensity of rhodamine 123 (Rh123). Yeast cells were first incubated in YPGE with the indicated concentration of ART for 30 min, then 2 µM Rh123 was used to incubate cells for 30 min at 30°C before washing and resuspension in PBS. The membrane potential of yeast cells was determined using a flow cytometer (FACScan, BD), with the excitation at 480 nm and emission at 530 nm. For each sample, 20,000 events were counted at the same flow cytometer setting.

### Oxygen consumption

After grown to OD 1.00, yeast cells were treated with ART or DHA for 30 min, and then the cells were centrifuged, washed and resuspended by PBS. Oxygen consumption was measured using a Clark-type oxygen electrode in a total volume of 1 ml.

### ART content analysis 

Liquid chromatography-mass spectrometry (LC-MS) was applied to detect the ART in samples. HPLC was performed using an Agilent 1290 system (Palo Alto, CA, USA). The HPLC system was coupled to an Agilent 6460 triple-quadrupole mass spectrometer (Palo Alto, CA, USA) via a electrospray ionization (ESI) interface for mass analysis and detection. Chromatographic separation was achieved on an Acquity UPLC^®^ BEH C18 column (2.1 × 50 mm, 1.7 μm; Waters Corp., Milford, USA ). SecurityGuard C18 (5 µm) guard column (Waters Corp., Milford, USA). The chromatography was performed at room temperature. The mobile phase consisted of acetonitrile/ water containing 0.1% (v/v) formic acid (30:70, v/v), delivered at a flow rate of 0.2 mL/min. The mass spectrometer was operated in positive ion mode. The fragmentation transitions for the multiple reactions monitoring (MRM) were m/z 283.0 to 247.0 for ART.

For sample preparation, yeast cells were incubated with ART in YPGE medium for 20 min before centrifugation and the supernatant was collected and then extracted with 3 vol of dichloromethane for 3 times. The samples were air dried and resolved by 30% methanol before injection and chromatographic analysis.

### Cytochrome *c* oxidase activity assay 

Activities of cytochrome *c* oxidase were measured by the Cytochrome *c*-Oxidase Assay kit (# CYTOCOX1, Sigma, St. Louis, MO, USA) according to manufacturer's instructions.

### Statistical analysis

Results are expressed as mean ± SD for the indicated number of experiments. Statistical analyses between parallel groups were calculated with Student’s t-test. A value of P under 0.05 was considered to be statistically significant.
